# Solar Ultraviolet Radiation Exposure Among Opencast Miners in Namibia with the Use of Electronic Dosimeters: A Feasibility Study

**DOI:** 10.5334/aogh.4490

**Published:** 2024-11-27

**Authors:** Motsehoa Cynthia Ramotsehoa, Frederik Christoffel Eloff, Johannes Lodewykus du Plessis, Caradee Yael Wright, David Jean du Preez

**Affiliations:** 1 Occupational Hygiene & Health Research Initiative, Faculty of Health Sciences, North‑West University, Potchefstroom, South Africa; 2 Environmnetal and Health Research Unit, South African Medical Research Council (SAMRC), Pretoria, South Africa; 3 Department of Geography, Geoinformatics and Meteorology, University of Pretoria, Pretoria, South Africa; 4 Department of Environmental Health, University of Johannesburg, Johannesburg, South Africa; 5 FORRS Partners GmBH, Germany

**Keywords:** solar ultraviolet radiation, sun exposure, outdoor workers, occupational exposure

## Abstract

*Importance:* The lack of information on exposure of opencast mineworkers to solar ultraviolet radiation, a group I carcinogen, was addressed. The feasibility of using electronic dosimeters in the determination of exposure to solar ultraviolet radiation was investigated.

*Objective:* The objective of the study was to determine the feasibility of measuring the occupational exposure of opencast mineworkers to solar ultraviolet radiation using electronic dosimeters.

*Design:* The study followed a cross‑sectional design.

*Setting:* Measurements were carried out at two opencast diamond mining operations hereafter referred to as site A and B, located in the Karas region of Namibia.

*Participants:* Workers from all four outdoor occupations (bedrock, engineering, metallurgy and security) were recruited to participate in the study.

*Measurements:* The study was conducted over four days at each site during winter (site A: 28 June to 4 July 2018 and site B: 6–11 July 2018) in the Karas region of Namibia with 28 consenting workers taking part. The AlGaN photodiode‑based electronic dosimeters were worn above clothing on the dorsal wrists (one) and two placed on the horizontal, unshaded area from 08:00 to 16:00 for the measurement of personal and ambient solar ultraviolet radiation, respectively. Historical meteorological data for the measurement period were obtained from Solcast and Ozone Monitoring Instrument (OMI) NASA.

*Results:* Overall, clear skies and surface reflectivity of 0.19 were observed for both study sites. The mean ultraviolet indices were 2.43 (0.06–4.51) and 2.24 (0.09–4.88) for site A and B, respectively. Findings of valid measurements from nine participants showed the mean total daily personal solar ultraviolet radiation exposure of 1.9 ± 1.0 (1.01–1.57) standard erythemal dose (SED) for site A and 3.4 ± 2.6 (3.39–7.28) SED for site B.

*Conclusions and Relevance:* Personal solar ultraviolet radiation exposure above the occupational exposure limit (OEL) demonstrated the need to include the winter season in planning for protective measures for skin and eyes, since workers are at risk of excessive exposure to solar ultraviolet radiation.

## Background

This study focused on the occupational exposure to solar ultraviolet radiation of mineworkers in opencast (also known as open pit) diamond mining operations in Namibia. According to the most recent definition [1], opencast mineworkers are typical outdoor workers since they spend more than 22% of their work shift outdoors. Occupational exposure to solar ultraviolet radiation, a group I human carcinogen linked to skin cancer [2], and negative effects on the eyes [3], represents a neglected health risk for outdoor workers [4]. The lack of an occupational exposure limit (OEL) for solar ultraviolet radiation exposure in African countries, including Namibia [5, 6] exacerbates the plight of exposed workers. Overall, the proportion of occupationally exposed individuals in Namibia stood at 26.86% (95% uncertainty range 22.72–31%) in 2019, while the population attributable percentage of deaths due to occupational solar ultraviolet radiation exposure‑related non‑melanoma skin cancer (NMSC) is 34.12% (26.23–43.51%) [7]. In Africa, cases of cataracts accompanied by vision impairment and cataract blindness are estimated to increase to 32 million and 8 million, respectively, by 2030 [8]. Thus, exposure to solar ultraviolet radiation and subsequent occupational health risk of approximately 9000 employees, who played a vital role in contributing towards the 14% contribution to the gross domestic product of Namibia’s mining industries at the time of the study [9], are not getting the attention that is warranted.

Therefore, this study aimed to explore the feasibility of measuring occupational exposure of opencast mineworkers to solar ultraviolet radiation in two mining chamber operations in Namibia using electronic dosimeters.

## Methods

### Ethical considerations

Ethics approval for this cross‑sectional study was received from the Namibia Ministry of Health and Social Services (reference: 17/3/3/CR) and the Health Research Ethics Committee of the North‑West University, South Africa (reference number: NWU‑00031‑17‑A1). The researcher presented the study objectives, methods, confidentiality and voluntary participation aspects to the workers. An independent person, trained in ethics, was also introduced to the workers and from whom they obtained consent forms and returned signed consent forms. The completed informed consent forms were kept in a locked cabinet in the administration building until the end of measurements. Subsequently, all forms are kept in the study leader’s office in a locked cabinet where they will remain for 10 years. For reporting purposes, data are anonymised using codes.

### Study sites

Measurements were carried out at two opencast mining operations, hereafter referred to as site A (latitude: 28°10′16″ S; longitude: 16°52′1″ E) and site B (latitude: 28°15′47″ S; longitude: 16°48′12″ E) located within the Karas region in the southernmost part of Namibia.

### Participants

Workers in all outdoor occupations were recruited to take part in the study. The outdoor occupations at site A were classified as bedrock, metallurgy, engineering and security officers. Except for bedrock, the same occupations were found at site B. The bedrock workers prepared the bedrock for blasting and cleared the rocks to create paths for vehicles. Metallurgy workers were responsible for cleaning spills and ensuring that the conveyor belts were running properly, while maintenance work, such as welding, was the responsibility of engineering workers. Security officers were responsible for access control to the mining sites.

### Measurements

The meteorological variables for the measurement period, namely cloud opacity, precipitation rate and albedo, were obtained from Solcast [10] and the total ozone column obtained from Ozone Measuring Instrument (OMI) [11]. The ultraviolet index (UVI) was calculated as a function of the zenith solar angle (SZA) and total ozone column (TOC) using the equation 1:

$$UVI\sim 12.5\;{cosSZA}^{2.42}\;{\left(TOC/300\right)}^{-1.23}$$ Equation 1

[12]

Electronic dosimeters used in this study to measure ambient and personal solar ultraviolet radiation exposure were designed and produced by Professor Martin Allen (University of Canterbury and MacDiarmid Institute for Advanced Materials and Nanotechnology). These dosimeters (Supplement Figure 1) measure erythemally weighted solar ultraviolet radiation exposure with the aid of two types of aluminium gallium nitride (AlGaN) Schottky photodiodes enclosed within a polytetrafluoroethylene (PTFE) casing, as a sensing unit [13, 14].

Study measurements were done over a four‑day period during weekdays in the winter season from 28 June to 4 July 2018 for site A and 6–11 July 2018 for site B. To quantify the amount of solar ultraviolet radiation reaching the ground in the absence of shade (i.e., ambient exposure), two electronic dosimeters were placed on a flat, horizontal surface. Also, workers wore one electronic dosimeter each, placed above clothing on the dorsal wrist of the dominant arm using fabric wristwatch straps for the measurement of personal solar ultraviolet radiation exposure. The dosimeters we set to take measurements at one‑minute intervals from 08:00 to 16:00.

For calibration, all electronic dosimeters used in the study were placed next to the Solar Light 501 UV Biometer sensor located on the roof of a building at the South African Weather Services (SAWS) in Irene, Pretoria, South Africa (latitude 25.91° S; longitude 28.21° E; altitude 1524 m). The dosimeters were set to measure solar ultraviolet radiation in counts between 08:00 and 16:00 at one‑minute intervals for seven days. We summed the one‑minute counts to ten minutes to match the Biometer measurement intervals. The Biometer data in minimal erythemal dose (MED) were converted to the standardised unit of erythemal effective radiation exposure, that is, the standard erythemal dose (SED), where 1 SED = 100 J/m [15]. Equations generated from the dosimeter calibration curves were then used to calculate the SED value equivalent to the electronic dosimeter counts. To remove variability and improve the calibration equations, only data from clear sky days were used in the calculations [13, 16].

Basic descriptive statistics in the form of mean, standard deviation (SD) and range were used in the description of meteorological data, total daily exposure, variations in total daily exposure, exposure by occupation, as well as a percentage of ambient solar ultraviolet radiation exposure. Statistical analyses and visualisation of data were performed in GraphPad Prism version 9.5.0.730 for Windows (GraphPad Software, San Diego, California, USA).

## Results

Historical satellite meteorological data [10, 11] (Table 1) for the two measurement sites show clear skies with no rainfall, except at the start of the measurements on 29 June at site A when cloud opacity was at 29% at 08:00, 5.5% at 09:00 and 0% for the rest of the day. Surface reflectivity of 0.19 was observed throughout the measurement period at both sites. The daily mean ultraviolet indices were 2.43 and 2.24 with a maximum of 4.51 and 4.88 at noon for site A and B, respectively.

**Table 1:** Meteorological Factors During the Study Period.

VARIABLE (UNIT)	SITE A MEAN (RANGE WHERE APPLICABLE)	SITE B MEAN (RANGE WHERE APPLICABLE)
Cloud opacity (%) [10] Precipitation rate (mm/h) ^10^Surface reflectivity [10] Calculated ultraviolet index [12]	0.9 (0–29) 0 0.19 2.43 (0.06–4.51)	0 0 0.19 2.24 (0.09–4.88)

The nine participants whose measurements were valid comprised five workers from the following occupations at site A, namely bedrock (*n* = 3), metallurgy (*n* = 1) and engineering (*n* = 1) and four workers at site B, which were represented by the following occupations, namely security (*n* = 1) and metallurgy (*n* = 4). In terms of gender, there were eight males and one female with an average age of 43 years (22–59 years), who classified themselves as Black Africans (Ramotsehoa *et al.*, 2024 unpublished).

### Ambient and personal solar ultraviolet radiation exposure data

**Figure 1:**
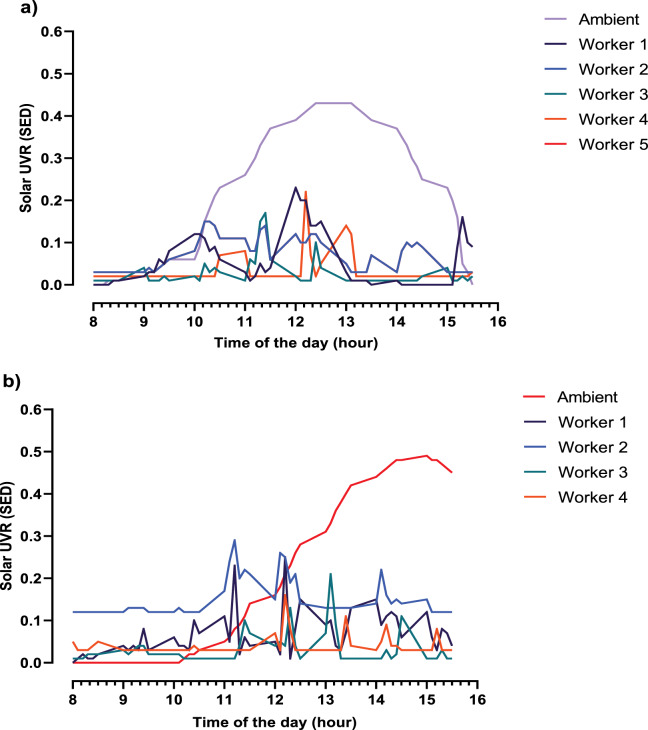
Ambient and personal solar ultraviolet radiation diurnal variations for **(a)**, Site A and **(b)**, Site B.

The solar ultraviolet radiation exposure measurements in the form of means and means ± standard deviation (range) are presented in Table 2 and Figure 1 in terms of measurement site, worker and occupation.

**Table 2:** The mean total daily solar ultraviolet radiation exposure.

SITE	SOLAR ULTRAVIOLET RADIATION EXPOSURE MEASUREMENT TYPE	OCCUPATION	VALID MEASUREMENT DAY(S)	TOTAL AND TOTAL MEAN DAILY SOLAR ULTRAVIOLET RADIATION EXPOSURE ± SD (SED)	SOLAR ULTRAVIOLET RADIATION EXPOSURE AS A PERCENTAGE OF AMBIENT (%)
A (n = 5)	Ambient	–	1, 2, 3	10.5 ± 0.7	–
	Worker 1	Bedrock	1, 3	2.7 ± 1.8	25.6 ± 17.1
	Worker 2	Bedrock	1, 2, 3	3.3 ± 1.4	32.1 ± 13.4
	Worker 3	Bedrock	3, 4	1.3 ± 0.0	13.1 ± 0.1
	Worker 4	Metallurgy	3	1.4	13.6
	Worker 5	Engineering	3	1.01	9.5
	*Worker average*			1.9 ± 1.0	18.8 ± 9.5
B (n = 4)	Ambient	–	2, 3	10.2 ±1.4	–
	Worker 1	Security	4	3.2	31.6
	Worker 2	Metallurgy	2, 3	7.2 ± 0.6	71.1 ± 6.0
	Worker 3	Metallurgy	2, 3	1.5 ± 0.1	15.3 ± 1.1
	Worker 4	Metallurgy	2	1.8	18.4
	*Worker average*			3.4 ± 2.6	34.1 ± 25.6

The mean daily ambient solar ultraviolet radiation for site A was 10.5 ± 0.7 (9.69–11.07) SED. The ambient solar ultraviolet radiation followed a typical bell shape with levels that peaked between 12:00 and 13:00 (Figure 1a). The mean daily personal exposure for site A was 1.9 ± 1.0 (1.01–3.39) SED (Table 2). The mean total daily solar ultraviolet radiation exposure for bedrock workers (n = 3) was 2.5 ± 1.0 (1.39 to 3.39) SED, while the daily solar ultraviolet radiation for the metallurgy worker (*n* = 1*)* and for the engineering worker (*n* = 1) was 1.4 SED and 1.0 SED, respectively. The corresponding mean daily solar ultraviolet radiation exposure as a percentage of ambient was 18.8 ± 9.5% (9.5 to 32.1%).

For site B, the mean daily ambient solar ultraviolet radiation was 10.2 ± 1.4 (3.39–7.28) SED. The ambient solar ultraviolet radiation levels showed an increase from 10:00 and peaked between 14:00 and 15:00 (Figure 1b). The mean daily personal solar ultraviolet radiation exposure for site B was 3.4 ± 2.6 (1.5–7.2) SED, while daily solar ultraviolet radiation exposure for the security officer (*n* = 1) was 3.2 SED and for metallurgy workers (*n* = 3) was 3.5 ± 3.2 (1.8–7.2) SED. The mean daily solar ultraviolet radiation exposure as a percentage of ambient was 34.1 ± 25.6% (15.34–71.1%).

## Discussion

To our knowledge, this is the first study in which personal solar ultraviolet radiation exposure amongst opencast mineworkers in Namibia was measured.

The peak ambient solar ultraviolet radiation level occurred between 12:00 and 13:00 (2.9 SED), in line with solar noon in Namibia at around 12:45, resulting in a characteristic bell‑shaped curve for site A. Site B’s ambient solar ultraviolet radiation peaked between 14:00 and 15:00 (3.2 SED), a deviation from the expected solar noon which may have been due to the winter season and locality [17].

Of significance for this study was that the mean daily solar ultraviolet radiation exposure for both measurement sites exceeded the International Commission on Non‑Ionizing Radiation Protection (ICNIRP) Occupational Exposure Limit (OEL) range of 1.0–1.3 SED, aimed at the prevention of acute health effects of the skin and eyes [15]. More specifically, the guideline is to be treated as a ceiling limit for the eyes and a goal to strive towards for the skin [18]. Similarly, the wintertime mean daily solar ultraviolet radiation exposures that exceeded the ICNIRP–OEL were reported in a study of South African outdoor workers (upper arms, 5.4 SED) (Hadjee, 2020 unpublished data, https://repository.nwu.ac.za/handle/10394/26476/discover.) and New Zealand outdoor [19] workers (4.8 SED). Given that these were wintertime exposures, the summertime exposure levels are likely to be higher [20]. Inversely, the mean personal solar ultraviolet radiation exposure measured on the shoulder using polysulphone dosimeters was higher in winter than in summer for Australian teachers [21]. Furthermore, findings from a study conducted by Parisi *et al*. [22] showed an increase of 2.0 SED in the exposure of all participants during winter.

The mean solar ultraviolet radiation exposure as a percentage of ambient in this study was similar to those obtained in one of the pioneering studies by Larko and Diffey [23], while other studies reported lower (Hadjee, 2020 unpublished data, https://repository.nwu.ac.za/handle/10394/26476/discover) and higher [24] wintertime mean solar ultraviolet radiation as a percentage of ambient*.* Diurnal variations observed in personal solar ultraviolet radiation exposures (Supplement File 2) highlighted the importance of other variables that may require further investigation, such as the types of tasks performed, postures assumed and different work areas related to the proportion of the atmosphere (sky) that is not blocked by structures, as well as general human behaviour [18]. Also, the difference in findings from the two measurement sites highlights the importance of measuring outdoor worker exposure to solar ultraviolet radiation [25], even at sites that are within the same country region.

Noteworthy is that the layout in opencast mining sites does not allow for extensive control of environmental factors to protect against excess solar ultraviolet radiation exposure [26], and the only control measure observed at the study sites was shading provided during lunch breaks. This is significant because the combination of clear skies, the noon ultraviolet index in the moderate range (3–5), the high personal solar ultraviolet radiation exposure, the need for protective measures against solar ultraviolet radiation exposure such as seeking shade, covering the exposed skin and application of sunscreen are recommended [27]. Although the workers’ risk for developing skin cancer are lower due to their darker skin types, they are not immune to other negative health effects of the skin and eyes [20, 28]. Modenese *et al.* [29] recommends intentional risk evaluation for solar ultraviolet radiation exposure of the eyes and examination of eyes for outdoor workers already exposed, followed by the provision of task‑appropriate protection and health surveillance as part of the occupational health services. Additionally, tailor‑made, skin phototype‑specific health messages, policies and training on sun‑safe exposure practices are required [30, 31] for the improvement in the use of and compliance with sun protection measures [32].

## Limitations

Failure of the electronic dosimeters to transfer data and battery failure in this study presented a limitation to the measurements similar to those reported by Køster *et al*. [33] for the same type of electronic dosimeters. It is acknowledged that surface reflectivity will influence solar ultraviolet radiation exposure and that the dosimeters used would have been able to record it. However, we did not set out to measure surface reflectivity, and this presents a limitation of the study.

## Conclusion

Study findings demonstrate the need for adequate sun protection for the skin and eyes to include winter since cumulative exposure is an important aspect of outdoor workers’ occupational health. The feasibility of producing time‑stamped personal solar ultraviolet radiation exposure was demonstrated.

## Additional Files

The additional files for this article can be found as follows:

Supplementary File 1. Figure 1.

Supplementary File 2. Ethics approval letters.
