# Supplementary material for: Solar Ultraviolet Radiation Exposure Among Opencast Miners in Namibia with the Use of Electronic Dosimeters: A Feasibility Study
Source: Ann Glob Health. 2024 Nov 27;90(1):73. doi: 10.5334/aogh.4490 (PMC11606395; doi:10.5334/aogh.4490)
Supplement: Supplementary File 1. — Figure 1. [file agh-90-1-4490-s1.pdf]

SOLAR ULTRAVIOLET RADIATION AMONG OPENCAST MINERS WITH THE USE OF ELECTRONIC DOSIMETERS: A FEASIBILTY STUDY

Supplement 1

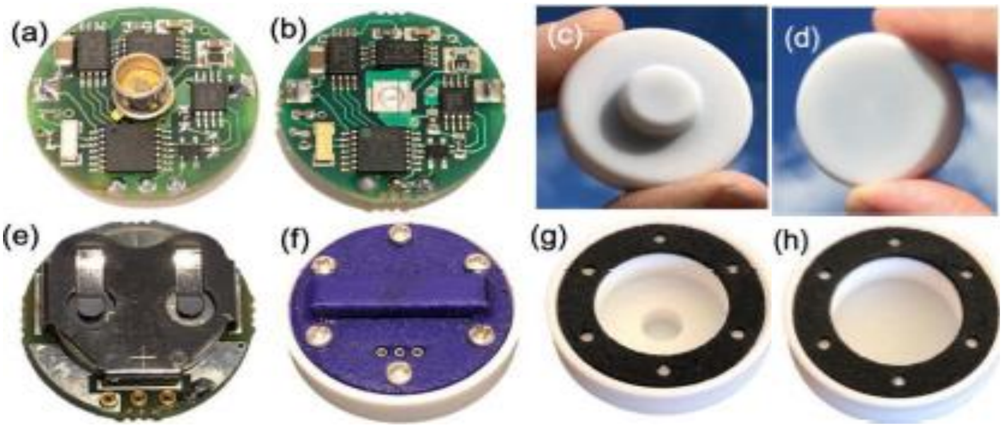

Figure 1. Electronic ultraviolet dosimeter construction: printed circuit board (pcb) incorporating (a) Type A and (b) Type B AlGaN photodiodes; PTFE diffuser caps for the (c) Type A and (d) Type B dosimeters; (e) pcb backside showing the CR1632 lithium-ion battery and holder, (f) nylon backing plate showing the 3-pin micro data port, inside of the (g) Type A and (h) Type B diffuser caps with neoprene gaskets.

(Reproduced with permission from Prof M. Allen).
