# Supplementary material for: Solar Ultraviolet Radiation Exposure Among Opencast Miners in Namibia with the Use of Electronic Dosimeters: A Feasibility Study
Source: Ann Glob Health. 2024 Nov 27;90(1):73. doi: 10.5334/aogh.4490 (PMC11606395; doi:10.5334/aogh.4490)
Supplement: Supplementary File 2. — Ethics approval letters. [file agh-90-1-4490-s2.pdf]

Supplement 2: Proof of ethics approval

2.1 Republic of Namibia Ethics approval certificate

REPUBLIC OF NAMIBIA

Ministry of Health and Social Services

Private Bag 13198 | Ministerial Building | Tel: 061 – 2032150
Windhoek | Harvey Street | Fax: 061 – 222558
Namibia | Windhoek | Email: shimenghipangelwa71@gmail.com

OFFICE OF THE PERMANENT SECRETARY

Ref: 17/3/3 **CR**
Enquiries: Mr. J. Nghipangelwa

Date: 15 December 2017

Ms. Cynthia Ramotsehoa
North West University
P.O. Box X6001, Potchefstroom
South Africa

Dear Ms Ramotsehoa

RE: Occupational exposure of workers to solar ultraviolet radiation at selected opencast mining operations in Namibia and Chile.

1. Reference is made to your application to conduct the above-mentioned study.
2. The proposal has been evaluated and found to have merit.
3. **Kindly be informed that permission to conduct the study has been granted under the following conditions:**

3.1 The data to be collected must only be used for academic purposes;
3.2 No other data should be collected other than the data stated in the proposal;
3.3 Stipulated ethical considerations in the protocol related to the protection of Human Subjects' should be observed and adhered to, any violation thereof will lead to termination of the study at any stage;
3.4 A quarterly report to be submitted to the Ministry's Research Unit;

3.5 Preliminary findings to be submitted upon completion of the study;

3.6 Final report to be submitted upon completion of the study;

3.7 Separate permission should be sought from the Ministry of Health and Social Services for the publication of the findings.

Yours sincerely,

Ms. P Masabane
Acting Permanent Secretary

2.2 North-West University Health Research Ethics Committee (HREC) of the Faculty of Health Sciences approval letter

NWU®
NORTH-WEST UNIVERSITY
NOORDWES-UNIVERSITEIT
YUNIBESITI YA BOKONE-BOPHIRIMA

Private Bag X6001, Potchefstroom
South Africa 2520

Tel: 018 299-1111/2222
Web: http://www.nwu.ac.za

Health Sciences Ethics Office for Research, Training and Support

North-West University Health Research Ethics Committee (NWU-HREC)
Tel: 018-299 2092
Email: Wayne.Towers@nwu.ac.za

Prof FC Eloff
Occupational hygiene
OHHRI

19 November 2021

Dear Prof Eloff

APPROVAL OF YOUR AMENDMENT REQUEST BY THE NORTH-WEST UNIVERSITY HEALTH RESEARCH ETHICS COMMITTEE (NWU-HREC) OF THE FACULTY OF HEALTH SCIENCES

Ethics number: NWU-00031-17-A1

Kindly use the ethics reference number provided above in all future correspondence or documents submitted to the administrative assistant of the North-West University Health Research Ethics Committee (NWU-HREC) secretariat.

Study title: Occupational exposure of workers to solar ultraviolet radiation at selected opencast mining operations in Namibia

Study leader/Researcher: Prof FC Eloff

Student: MC Ramotsehoa - 10074031

You are kindly informed that your amendment request (changes to research sites to be used) to the aforementioned project has been approved. Any future amendments to the proposal or other associated documentation must be submitted to the NWU-HREC, Faculty of Health Sciences, North-West University, prior to implementing these changes. These requests should be electronically submitted to Ethics-HRECApply@nwu.ac.za, for review BEFORE approval can be provided, with a cover letter with a specific subject title indicating, "Amendment request: NWU-XXXXX-XX-XX". The letter should include the title of the approved study, the names of the researchers involved, the nature of the amendment/s being made (indicating what changes have been made as well as where they have been made), which documents have been attached and any further explanation to clarify the amendment request being submitted. The amendments made should be indicated in yellow highlight in the amended documents. The *e-mail*, to which you attach the documents that you send, should have a *specific subject line* indicating that it is an amendment request e.g. "Amendment request: NWU-XXXXX-XX-XX". This e-mail should indicate the nature of the amendment. This submission will be handled via the expedited process.

Please note: Due to the nature of the amendment i.e. (data will no longer be collected from the South American site and has already been collected from the Namibian site), this study will be able to proceed during the current alert level, following receipt of the approval letter. No additional COVID-19 restrictions have been placed on the study except that the researcher must ensure that before proceeding with the study that all research team members have reviewed the North-West University COVID-19 Occupational Health and Safety Standard Operating Procedure.

We wish you the best as you conduct your research. If you have any questions or need further assistance, please contact the Faculty of Health Sciences Ethics Office for Research, Training and Support at Ethics-HRECApply@nwu.ac.za.

Yours sincerely

Digitally signed by
Prof Petra Bester
Date: 2021.11.23
11:11:25 +02'00'

Chairperson: NWU-HREC

Current details: (23239522) G:\My Drive\9. Research and Postgraduate Education\9.1.5.3 Letters Templates\9.1.5.4.1_Approval_letter_Amend_Req_HREC.docm
30 April 2018

File reference: 9.1.5.4.1
